# The Impact of Oncogenic Viruses on Cancer Development: A Narrative Review

**DOI:** 10.3390/biology14070797

**Published:** 2025-07-01

**Authors:** Maria Karoliny da Silva Torres, Gabriel dos Santos Pereira Neto, Izaura Maria Vieira Cayres Vallinoto, Leonardo Oliveira Reis, Antonio Carlos Rosário Vallinoto

**Affiliations:** 1 Laboratório de Virologia, Instituto de Ciências Biológicas, Universidade Federal do Pará, Belém 66075-110, Pará, Brazil; karolinytorres15@gmail.com (M.K.d.S.T.); gabriel.neto@ics.ufpa.br (G.d.S.P.N.); ivallinoto@ufpa.br (I.M.V.C.V.); 2 Programa de Pós-Graduação em Biologia de Agentes Infecciosos e Parasitários, Instituto de Ciências Biológicas, Universidade Federal do Pará, Belém 66075-110, Pará, Brazil; 3 UroScience, State University of Campinas, Unicamp, Campinas 13083-970, São Paulo, Brazil; reisleo@unicamp.br; 4 ImmunOncology, Pontifical Catholic University of Campinas, PUC-Campinas, Campinas 13087-571, São Paulo, Brazil

**Keywords:** cancer, virus, oncogenesis, oncoviruses, therapy, pathogenesis

## Abstract

Oncogenic viruses are infectious agents that can cause cancer in humans and animals. They are estimated to be responsible for approximately 12% of human cancers worldwide. These viruses trigger a series of mechanisms that allow them to insert their genetic material into host cells, disrupting normal cellular processes and leading to uncontrolled growth and tumor formation. This article reviews the literature on the main oncogenic viruses and reports on newly identified viruses potentially associated with cancer development, addressing the mechanisms of oncogenesis and the types of cancers associated. In addition, the article brings together the evidence for preventive strategies, such as vaccination, and therapeutic advances in combating oncogenic viral infections. This review discusses the role of early detection and treatment in managing virus-related cancers globally. This article reviews current prevention and treatment strategies, including HPV and HBV vaccines and antiviral therapies, and mentions future approaches like immunotherapies and CRISPR/Cas9. Therefore, this article underscores the importance of studying the dynamics of co-infection and the role of human microbiota in viral persistence and carcinogenesis, which opens new possibilities for combination therapies and microbiome-based interventions to slow the progression of viral-related tumors.

## Simple Summary

Oncogenic viruses are responsible for approximately 15–20% of human cancers worldwide. These viruses employ molecular mechanisms that alter cellular functions, leading to uncontrolled cell growth and tumor formation. This review highlights the major oncoviruses (HPV, HBV, HCV, EBV, KSHV, HTLV-1, and MCPyV) and their roles in cancer development. It explores the mechanisms of viral oncogenesis, the types of cancers involved, and preventive strategies such as vaccination. Overall, the review aims to summarize recent advances in understanding the virus–cancer relationship, offering new insights into oncogenic processes and perspectives for future research.

## 1. Introduction

The discovery that certain human viruses cause many cancers is a breakthrough in understanding and treating cancer. Over a century ago, Dr. Peyton Rous suggested that viruses could cause cancer by observing that filtered extracts of chicken fibrosarcoma could transmit the disease [1]. The correlation between Epstein–Barr virus (EBV) and Burkitt’s lymphoma subsequently expanded the understanding of the involvement of viruses in human carcinogenesis [2].

Recent estimates suggest that viral infections cause over 1.4 million cancer cases annually, accounting for about 15–20% of the global cancer burden [3]. The widely accepted human oncoviruses are human papillomavirus (HPV), hepatitis B virus (HBV), hepatitis C virus (HCV), Epstein–Barr virus (EBV), Kaposi’s sarcoma-associated herpesvirus (KSHV) (also called human herpesvirus 8), human T-cell lymphotropic virus (HTLV-1), and Merkel cell polyomavirus (MCPyV). This conclusion draws from numerous experimental, clinical, and epidemiological studies over the past fifty years [4].

According to the International Agency for Research on Cancer (IARC), in 2018, approximately 2.2 million cases of cancer globally were attributable to infectious agents, including bacteria, viruses, and parasites, with different geographical distribution between low- and high-income countries [5,6]. These viruses present diverse characteristics contributing to oncogenesis, with the infectious process being yet another factor in the multifactorial and complex process involving carcinogenesis.

## 2. Methods

This study is a narrative review aiming to map and discuss the mechanisms by which oncogenic viruses contribute to cancer development, integrating molecular, immunological, and epidemiological approaches. To ensure greater scientific rigor, methodological transparency, reproducibility, and reliability of the findings, we chose to adopt a structured scoping review approach, following the guidelines of the Preferred Reporting Items for Systematic Reviews and Meta-Analyses for Scoping Reviews (PRISMA-ScR) 2020 extension. The analysis of the studies was conducted in a narrative and descriptive manner, allowing the thematic synthesis of the available evidence.

### 2.1. Information Sources and Search Strategy

A comprehensive literature search was conducted across three electronic databases: PubMed, Scopus, and Web of Science. The search covered publications from January 2000 to March 2025. The following search terms and Boolean operators were used: “oncogenic virus” OR “cancer” AND “HPV” OR “HBV” OR “HCV” OR “EBV” OR “KSHV” OR “HTLV-1” OR “MCPyV” OR “viral oncogenesis” OR “virus-induced carcinogenesis” OR “epigenetics” OR “immune evasion” OR “inflammation” OR “microbiome and cancer”. Manual searches of the reference lists from key articles were also conducted to identify additional relevant studies.

### 2.2. Eligibility Criteria

The inclusion criteria were as follows: original research articles, systematic reviews, and meta-analyses; studies focused on viral oncogenesis mechanisms, pathogenesis, epidemiology, prevention, or treatment; and articles published in English, Portuguese, or Spanish.

The exclusion criteria were as follows: conference abstracts, editorials, commentaries, and letters to the editor and studies that did not explicitly address virus-associated cancer.

### 2.3. Selection of Sources of Evidence

After removing duplicates, two independent reviewers screened titles and abstracts for relevance. Potentially eligible articles were retrieved in full text for detailed assessment. Discrepancies were resolved through discussion and consensus with a third reviewer. The selection process is depicted in the PRISMA flow diagram, which is included in the Supplementary Materials.

### 2.4. Data Charting Process

Data from the included studies were charted using a standardized form developed by the authors. The form captured information on study type, virus studied, associated cancer(s), oncogenic mechanisms, and key findings. The data charting process was iterative, and the form was updated as new themes emerged during data extraction. A detailed critical analysis of each piece of evidence was performed, especially for studies proposing new associations between viral infections and cancer development.

This assessment considered multiple methodological aspects of the studies, such as sample size, robustness of the statistical analyses applied, study design (cross-sectional, case-control, cohort, case reports), laboratory techniques used for viral detection (PCR, sequencing, immunohistochemistry), and outcomes analyzed (presence of viral DNA/RNA in tumor tissues, expression of oncoproteins, evidence of genomic integration), as well as the potential impact of the findings. In addition, we sought to identify frequent methodological limitations, such as the absence of a control group, low statistical power, lack of replicability, or absence of functional studies that prove the oncogenic role of the virus.

### 2.5. Synthesis of Results

The synthesis was narrative and thematic. The information extracted was grouped and summarized by virus type, cancer type, mechanisms of oncogenesis, and emerging associations. Tables and figures were used to organize key data and enhance interpretability.

### 2.6. Compliance with PRISMA Guidelines

This review adhered to the PRISMA-ScR 2020 guidelines. The PRISMA-ScR checklist and flow diagram are provided in the Supplementary Materials.

## 3. Oncovirus: The Correlation Between Viral Infection and Cancer Development

Oncoviruses (OVs), or oncogenic viruses, are infectious agents that can trigger the development of cancer cells. Table 1 details the genomic and replication characteristics of the main families of oncogenic viruses capable of causing urogenital cancer, providing insights into their mechanisms of action and their role in oncogenesis.

HPV is a DNA virus of the *Papillomaviridae* family with tropism for squamous epithelial cells and the causative agent of warts and precancerous lesions that can eventually lead to the development of tumors of the cervix, anus, vagina, penis, oropharynx, vulva, oral cavity, and larynx [7]. Of the 448 types that have been documented, only 12 are currently classified as carcinogenic: types 16, 18, 31, 33, 35, 39, 45, 51, 52, 56, 58, and 597. HPV types 16 and 18 account for 70% of detectable HPV genotypes in cervical cancer.

Infections caused by cancerous types of HPV are very common; however, the immune system clears 80% within three years, and only 3% progress to cervical precancer/cancer within seven years [13]. When HPV infects target cells, HPV-derived oncogenes E6 and E7 inactivate the tumor suppressor genes p53 and pRb, respectively, leading to resistance to apoptosis and abnormal cell proliferation [14,15]. The development of cancer due to HPV is a rare consequence and not the main outcome of the infectious process.

Both EBV and KSHV belong to the Herpesviridae family and are also considered oncogenic viruses. HHV-4 has B-lymphocyte tropism, and EBV infection begins in the oropharynx, where it targets the oral mucosal epithelium and oropharyngeal B cells during primary infection [8,9]. The oropharyngeal mucosa is heavily infiltrated with lymphocytes, and cell-free viruses in saliva bind first to the cell membranes of the B lymphocytes, then to oropharyngeal epithelial cells. Once the lytic period of infection has passed, the virus establishes a long period of latency in memory B cells [9]. HHV-4 has been associated with several forms of cancer, including Burkitt’s lymphoma, B-cell lymphoma, nasopharyngeal carcinoma, and gastric cancer.

HBV and HCV use multiple mechanisms to bind to infected cells, which can unintentionally lead to the development of cancer, specifically hepatocellular carcinoma [10]. Common mechanisms used between these two viruses include: (1) persistent liver inflammation and immune-mediated oxidative stress damage from chronic viral infection; (2) intracellular oxidative stress damage induced by viral proteins; and (3) dysregulation of cellular signaling pathways by viral proteins (e.g., HBx, L-HDAg, S-HDAg, HCV core, NS3, and NS5A/B) [10]. HBV is the only hepatotropic DNA virus that also uses viral DNA integration to induce genome instability, which can lead to the creation of fusion gene products and altered expression of oncogenes or tumor suppressors. In addition, HCV facilitates metabolic reprogramming, leading to steatosis, which aids in the progression of fibrosis and hepatocellular carcinoma [10].

HTLV-1 is a member of the *Retroviridae* family that targets CD4+ T lymphocytes. HTLV-1 persists primarily in the infected host cell as a provirus and causes a malignancy called adult T-cell leukemia/lymphoma (ATLL) in about 5% of infections [16]. Tax and HBZ are currently recognized as two HTLV-1 oncogenes with distinct mechanisms. Tax can activate important cellular pathways, including those responsible for T-cell activation and expansion, and is therefore considered a key player in HTLV-1 persistence [17]. Central to its oncogenic activity is the abnormal, continuous activation of the NF-κB transcription factor family, which regulates cell proliferation, resistance to apoptosis, and immune responses. The Tax protein sequesters both pathways through direct protein–protein interactions. It binds IκBα and p105 via ankyrin-repeat domains, promoting IκBα degradation or complex dissociation, and thus, a persistent canonical signal. Simultaneously, Tax forms a complex with IKKα and p100, accelerating p100’s phosphorylation, ubiquitination and processing to p52 induced NF-κB activation. The result is chronic NF-κB activity, inflammatory cytokine production, and resistance to apoptosis [18,19].

This dual, Tax-mediated NF-κB activation underlies the clonal expansion of HTLV-1-infected T cells and contributes directly to HTLV-1-associated oncogenesis by creating a self-reinforcing loop of inflammation, survival signaling, and genetic instability [20].

HBZ is the only viral gene constitutively expressed during HTLV-1 infection, and its expression clearly demarcates infected from uninfected cells; the fundamental role of HBZ is to maintain HTLV-1 persistence by promoting infected cell survival [19].

MCV is a member of the *Polyomaviridae* family that exhibits tropism for epithelial cells. It is the causative agent of polyomavirus Merkel cell carcinoma, which is different than nonviral Merkel cell carcinoma produced by UV radiation [21]. In virus-positive cancers, MCV-dependent carcinogenesis requires two events. First, the viral genome is integrated into the host cell genome, followed by mutations in the gene encoding the large T protein, altering its DNA binding and helicase function domains, which renders viral replication incompetent [22]. The expressed T antigens then drive oncogenesis by altering multiple pathways and are required for the proliferation and survival of MCC cells [23].

### 3.1. Carcinogenesis

Cancer is a disease characterized by unregulated cell growth, with the potential for invasion and metastatic spread to other tissues and organs. This invasive and metastatic capacity results from a series of genetic and epigenetic alterations that compromise fundamental cellular pathways, including DNA repair mechanisms, cell cycle regulation, and evasion of the immune response [24,25].

The induction of cancer development can be divided into three main sections: primary, secondary, and favorable factors. The primary section highlights risk factors that can lead to infections. Radiation exposure, both ionizing and UV, is mentioned as an agent that can induce DNA mutations and is associated with the development of cancer, especially skin cancer [26]. In addition, risk factors such as family history, immunosuppression, and health behaviors, such as smoking, are fundamental, as they increase vulnerability to infection and cancer progression. The secondary section addresses genetic and biological aspects that contribute to oncovirus infection, associated with genetic alterations. Viral infection can also result in mutations that directly impact the cell cycle, affecting tumor suppressor genes and oncogenes, potentially leading to the development of cancer, for example, through the integration of the virus’s genetic material into the cellular genome, a critical mechanism that can cause deregulation of the cellular machinery, creating a microenvironment favorable to the development of a malignant neoplasm associated with viral infection [27]. Finally, in the favorable factors section, social and behavioral factors that can increase the likelihood of developing cancer are discussed, such as environmental and lifestyle factors, such as smoking and dietary habits [28].

Environmental carcinogenesis may be influenced by exposure to chemical agents that require metabolic activation, primarily mediated by cytochrome P450 (CYP) enzymes. This process can generate DNA lesions, which, if unrepaired, result in mutations that drive tumor initiation [29,30,31].

Neoplastic cells also undergo metabolic reprogramming to sustain rapid proliferation, notably via the Warburg effect, characterized by increased glucose uptake and aerobic glycolysis with lactate production [32,33]. Altered expression of transporters such as GLUT1 and repression of mitochondrial pyruvate transporters contribute to this phenotype and the formation of a tumor microenvironment (TME), which includes immune and stromal cells, vasculature, and extracellular matrix components [34,35].

Regulation of the cell cycle, particularly through cyclins and cyclin-dependent kinases (CDKs), is closely linked to both proliferation and metabolism. Dysregulation of CDKs, including CDK20 and CDK4/6, has been implicated in tumor progression and is a therapeutic target in various cancers [36,37,38]. Apoptosis evasion is another hallmark of cancer. Oncoviruses contribute to this by encoding proteins that inhibit apoptotic pathways and disrupt cell cycle regulation, thereby enhancing cell survival and promoting oncogenesis [39,40,41].

The mechanisms by which oncogenic viruses induce cancer are diverse and complex. They can directly interfere with the cell cycle, inhibit apoptosis, promote chronic inflammation, and modulate the tumor microenvironment to favor cell proliferation. The modulation caused by oncoviruses on the innate immune system, allowing immune evasion and persistence of viral infection, which includes the modulation of inflammatory responses and the inhibition of antiviral signaling pathways, facilitates oncogenesis [42].

### 3.2. Impact on the Cell Cycle

The cell cycle is a tightly controlled process that ensures correct cell division. This cycle consists of four main phases: G1, S, G2, and M. Progression through these phases is regulated by several proteins and protein complexes, including cyclins, CDKs, and CDK inhibitors. Abnormalities in cell cycle control, such as mutations in genes encoding cyclins, and dysregulations in the signaling cascade can lead to uncontrolled cell proliferation [43].

In the G1 phase, the first phase of the cycle, the cell grows and prepares for DNA replication. During the S phase, DNA synthesis takes place. The G2 phase is the second preparatory phase before mitosis. In the M phase, cell division occurs. During the cycle, there are two fundamental checkpoints, as highlighted in the diagram: the G1/S point, which regulates the transition from the G1 phase to the S phase, and the G2/M point, which controls the passage from the G2 phase to the M phase. These points are crucial to ensure that the cell is ready to advance in each cycle stage. During the cell cycle, oncogenes are genes that, when mutated or overexpressed, can promote cell division, potentially leading to cancer; they can also be activated during an infectious process [43]. Among these, cyclins stand out, which are proteins that regulate the cell cycle by activating CDKs [44]. CDKs are enzymes that, when activated by cyclins, direct the cell cycle forward. In addition, the transcription factor MYC, which stimulates cell proliferation, and the protein Mdm2, which regulates the tumor suppressor p53, are mentioned as oncogenes. On the other hand, tumor suppressor genes act by inhibiting cell division and preventing the formation of tumors. Important examples include the protein p53, which plays a crucial role in DNA repair and apoptosis (programmed cell death) [45]. Other proteins, such as p21, p27, and p57, are inhibitors of cyclin-dependent kinases, while p14 and p16 are also considered important regulators of the cell cycle [43].

Viral infections play a crucial role in oncogenesis, promoting malignant cellular transformation through diverse molecular mechanisms. Oncoviruses encode oncoproteins that interact with the host cellular machinery, leading to alterations in cell cycle regulation, genomic stability, and epigenetic gene expression, among other processes that favor uncontrolled cell proliferation and immune evasion [44]. The modulation of the cell cycle machinery favors viral replication and clonal expansion of infected cells [46]. These viral oncoproteins can deregulate cell cycle progression, promoting the activation of quiescent cells and their entry into the proliferative phase. Alterations in the expression of cyclins, CDKs, and CDK inhibitors result in the deregulation of cellular checkpoints, favoring oncogenesis [41,42,43,44].

**Figure 1:**
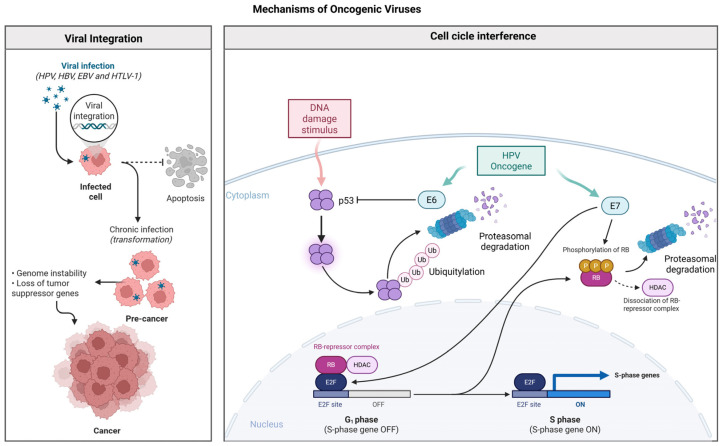
(**Left**) Viral integration into the host genome, as observed in HPV, HBV, EBV, and HTLV-1, can lead to chronic infection, genomic instability, and the disruption of tumor suppressor genes, favoring malignant transformation. (**Right**) In HPV-driven carcinogenesis, the E6 oncoprotein promotes proteasomal degradation of p53 via ubiquitination, impairing DNA damage response and apoptosis. Concurrently, E7 inactivates the retinoblastoma protein (pRb), releasing E2F transcription factors and enabling uncontrolled progression to the S phase. Both proteins contribute to cell cycle deregulation and tumor development through interference with chromatin regulators such as HDACs. Created using BioRender.

Tumor virus infection can interfere with mitotic checkpoints, resulting in immortalization and uncontrolled proliferation of infected cells. This phenomenon occurs through functional inhibition or proteasomal degradation of tumor suppressor proteins, facilitated by viral gene products. Removal of these mitotic control mechanisms is an essential factor for neoplastic progression [44,45,46,47] (Figure 1).

Integration of the viral genome into the host DNA can cause chromosomal rearrangements, leading to dysregulation of gene expression and loss of tumor suppressor genes such as TP53, causing genomic instability characteristic of oncovirus-associated neoplasms [48]. Furthermore, some viral oncoproteins modulate the expression of microRNAs (miRNAs) associated with genomic instability, worsening the process of cellular transformation [49,50] (Figure 1).

The correlation between infection by oncogenic viruses and epigenetic alterations that modulate host gene expression may also favor the development of neoplasia; these alterations include hypermethylation of tumor suppressor gene promoters and dysregulation of the expression of critical transcription factors [51]. Viruses such as HPV, EBV, HBV, and HCV can downregulate E-cadherin through overexpression of DNA methyltransferases (DNMTs), promoting aberrant methylation of the CDH1 gene and contributing to tumor progression [52,53,54]. The infectious process’s manipulation of several cellular signaling pathways leads to signal transduction via viral oncoproteins. The Notch signaling pathway, for example, is targeted by all DNA tumor viruses, regulating critical decisions about cell differentiation and proliferation [55]. Dysregulation of cellular signaling pathways, such as MAPK/ERK and PI3K/AKT, plays a crucial role in oncogenesis. Several genetic, biochemical, environmental, and behavioral factors can induce changes in these signaling pathways acting as oncogenic agents [56].

Prolonged exposure to ionizing radiation, chemicals, and infectious agents significantly increases cancer risk. OVs are a subgroup of viruses that induce cancer due to their potential to integrate into the host genome, modulate the immune response, and generate changes in cell physiology, leading to the activation of viral oncogenes or the inactivation of tumor suppressor genes [57]. EBV, HPV, and HTLV-1 are associated with several types of cancer, including cervical carcinoma, lymphomas, and leukemias [58].

OVs can interfere with the cellular cycle in many ways, promoting uncontrolled proliferation and favoring oncogenesis. Many viral oncoproteins inactivate tumor suppressors such as P53 and RB, allowing the cell to advance through cell cycle phases without proper repairs or verification [59,60].

Apoptosis can also be interfered with, leading to uncontrolled cell growth and tumor progression. The HPV E7 protein, for example, inhibits the pRb protein, an important cell cycle regulator, contributing to uncontrolled cell proliferation [61].

In the G1 phase, viruses like HPV use oncoproteins E6 and E7 to degrade P53 and inactivate RB, removing the brakes from cellular progression. As a result, infected cells continue to divide, even when there is DNA damage [62]. The E6 protein, by binding to E6AP (E6-associated protein), promotes the ubiquitination and degradation of p53, inhibiting the response to DNA damage and favoring the survival of genetically unstable cells. In addition, E6 activates the expression of telomerase (hTERT), allowing cell immortalization, a fundamental step in carcinogenesis. E6 also interferes with components of the DNA repair pathways, such as the nucleotide excision repair (NER) and base excision repair (BER) pathways, exacerbating genomic instability [59,62]. In turn, the E7 protein promotes the degradation of the retinoblastoma protein (pRb), releasing the E2F transcription factors, which activate the transcription of genes necessary for the progression of the S phase of the cell cycle, independently of the normal regulatory signals. E7 also interferes with chromatin structure through the modulation of enzymes such as histone deacetylases (HDACs) and remodelers such as p300/CBP, affecting gene expression epigenetically [61] (Figure 1).

During the S phase, viruses such as EBV and HTLV-1 can modulate the expression of growth factors and kinases involved in DNA replication, accelerating this process and increasing the chance of mutations [63,64]. In the G2 phase, viruses such as HBV and HCV can disrupt DNA repair mechanisms, allowing errors to be passed on to daughter cells. This generates genomic instability, which is one of the hallmarks of oncogenesis [65,66]. During mitosis, viruses such as KSHV (HHV-8) can influence the dynamics of the mitotic spindle, promoting aneuploidy and altering the expression of cell cycle regulatory genes, increasing the risk of neoplastic transformation [67].

### 3.3. Viral Infection and Cell Transformation

Different viruses have distinct mechanisms of carcinogenesis and can interfere with proto-oncogenes, tumor suppressor genes, and intracellular signaling pathways (Table 2). Thus, the integration of the viral genome into the host cell’s DNA is a crucial event in viral oncogenesis. This integration can cause mutations, genomic instability, and recombination, increasing genetic variability and accelerating tumor progression. HTLV-1, for example, integrates into the genome of T cells, causing mutations that promote cellular alterations and contribute to the development of ATLL [68]. Although oncoviruses share elements and signaling pathways, it is crucial to highlight the different oncogenic mechanisms that these viruses present, as demonstrated in Table 2.

Through mechanisms such as DNA methylation, histone modification, and microRNA expression, these viruses alter the epigenome of the host and the virus itself, directly impacting the characteristics of the cancer. A classic example is the integration of the HPV genome into the host DNA, resulting in epigenetic alterations that silence tumor suppressor genes, such as p53 and pRb, and promote tumor progression [69].

Alteration of gene expression mediated by viral oncoproteins and untranslated viral RNAs plays a fundamental role in modulating host cell gene expression. These viral molecules interfere with several stages of gene expression, from transcription and translation to protein stability, leading to uncontrolled cell proliferation and malignant transformation. HCV, for example, expresses the NS5A protein, which inhibits apoptosis and promotes angiogenesis, contributing to the development of hepatocellular carcinoma [70,71].

MiRNAs play a crucial role in regulating gene expression. Oncogenic viruses can encode their miRNAs (v-miRNAs) or modulate the expression of host miRNAs, impacting a wide range of genes and influencing the carcinogenic process [72]. Studies demonstrate that EBV encodes miRNAs that regulate the expression of genes involved in apoptosis and the immune response, contributing to cellular immortalization and immune evasion [73].

### 3.4. Inflammation

Chronic inflammation is a biological process that occurs when the immune system remains activated for prolonged periods. Persistent infections, autoimmune diseases, or continued exposure to irritants can cause this inflammatory state [74,75]. Unlike acute inflammation, which is a rapid and self-limiting response, chronic inflammation leads to the sustained production of pro-inflammatory cytokines, reactive oxygen species (ROS), and growth factors that favor cell survival and proliferation. This inflammatory environment contributes to genomic instability and may result in neoplastic transformation [76].

**Figure 2:**
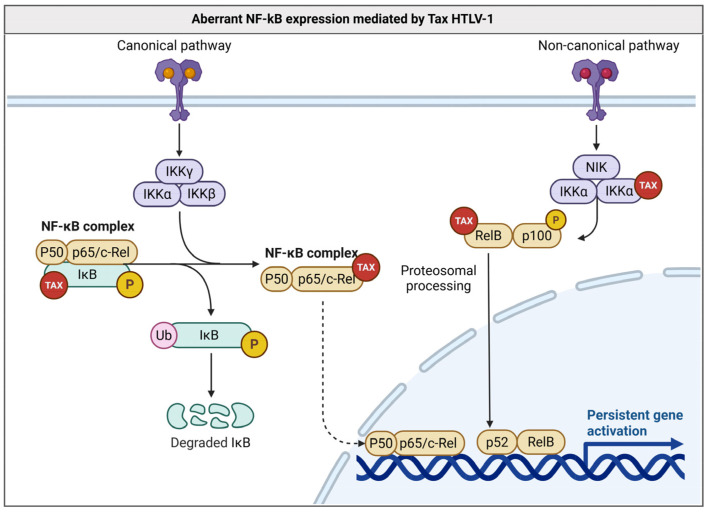
Constitutive activation of the canonical and non-canonical NF-κB signaling pathways promoted by the viral protein Tax. In the canonical pathway, Tax interacts with the IKK complex (IKKα, IKKβ, IKKγ), promoting the phosphorylation, ubiquitination, and subsequent degradation of IκB, releasing the NF-κB complex (p50/p65/c-Rel) for nuclear translocation and gene activation. In the non-canonical pathway, Tax stimulates the activation of NIK and IKKα, promoting the phosphorylation of the p100 protein bound to RelB. This leads to the proteasomal processing of p100 into p52, allowing the formation of the RelB/p52 complex, which also migrates to the nucleus and activates the transcription of target genes. Both pathways converge at the persistent activation of pro-proliferative and anti-apoptotic genes, which is characteristic of HTLV-1-induced cellular transformation. Created using BioRender.

Several signaling pathways play a central role in the regulation of chronic inflammation. Among them, the nuclear factor kappa B (NF-κB) pathway stands out, which controls the expression of inflammatory genes and is frequently activated in tumor cells [77]. Another relevant pathway is JAK/STAT, which is essential for cytokine signaling and uncontrolled cell growth [78]. In addition, the NLRP3 inflammasome pathway can promote the secretion of pro-inflammatory interleukins, such as IL-1β and IL-18, exacerbating the inflammatory process [79]. Thus, chronic inflammation is a process frequently exploited by OVs to create a microenvironment favorable to tumor growth. These viruses modulate the tumor microenvironment, promoting cell proliferation, angiogenesis, and the antitumor immune response suppression (Figure 2).

EBV and HTLV-1 are known to modulate the NF-κB pathway to promote the survival of infected cells, resulting in lymphocyte proliferation and predisposing the host to the development of lymphomas [80,81]. Similarly, KSHV/HHV-8 expresses viral proteins, such as vFLIP, that constitutively activate NF-κB, ensuring the evasion of apoptosis and promoting angiogenesis [82]. HPV interferes with the inflammatory response through the degradation of p53 and activation of the PI3K/AKT pathway, resulting in a chronic inflammatory microenvironment that favors the development of cervical carcinomas and other associated tumors [83,84].

The persistent inflammation promoted by these viruses creates a scenario in which cell proliferation is continuously stimulated, DNA repair mechanisms are inhibited, and the immune system is suppressed, allowing tumor progression. In addition, oncogenic viruses have developed sophisticated mechanisms such as cytokine and chemokine dysregulation to evade immune system surveillance, allowing infection to persist and facilitating oncogenesis [85].

### 3.5. The Role of Immunocompetent Cells in Virus-Induced Inflammation and Oncogenesis

Chronic inflammation induced by oncogenic viruses involves multiple immune cell types that contribute to the progression from persistent infection to malignant transformation.

Macrophages exhibit remarkable plasticity, differentiating along a spectrum from pro-inflammatory M1 phenotypes—characterized by high glycolytic metabolism and the secretion of TNF-α, IL-1β, IL-6, and IL-12—to immunoregulatory M2 states that rely on oxidative phosphorylation and produce IL-10 and TGF-β, contributing to tissue repair and resolution of inflammation [86]. This dynamic polarization is orchestrated by environmental cues such as IFN-γ, IL-4, IL-13, hypoxia, and metabolite availability and mediated through key signaling pathways including JAK–STAT (STAT1 versus STAT3/STAT6), NF-κB, and PI3K–Akt, as well as inhibitory receptors like PD-1 and TIM-3 [87,88].

Oncoviruses have evolved sophisticated strategies to exploit macrophage plasticity for immune evasion and the establishment of chronic infection. During the early phase of infection, pathogen-associated molecular patterns (PAMPs) often trigger an M1-like antiviral response that restricts viral replication [89]. However, many viruses subsequently induce M2 skewing by upregulating PD-L1, secreting viral interleukin-10 homologs, or modulating host cytokine networks [89]. In vitro studies demonstrate the HCV E2 protein promotes IL-10 production via STAT3 activation, thereby dampening cytotoxic immunity [90]. Viral proteins such as hepatitis B virus X protein can also directly abrogate type I interferon signaling and suppress the pro-inflammatory cytokine community, while cytopathic depletion of M1 macrophages further skews the tissue environment toward an immunosuppressive state [91].

In the context of chronic inflammation, macrophages play a dualistic role in oncogenesis. Persistent M1 activation may lead to a cytokine storm, generating elevated levels of reactive oxygen and nitrogen species that inflict DNA damage on resident cells [92]. As lesions become established, macrophages transition toward an M2-like tumor-associated macrophage (TAM) phenotype, creating a microenvironment conducive to malignant transformation and tumor progression. This intricate interplay between macrophage polarization and viral modulation underscores the central contribution of these innate immune cells to virus-induced inflammation and oncogenesis [93,94].

OVs have coevolved strategies to subvert NK cell surveillance by manipulating ligand–receptor interactions and intracellular signaling. In chronic HCV infection, hepatocytes upregulate Qa-1 (murine homolog of HLA-E), which engages NKG2A on NK cells through direct contact, triggering a sustained inhibitory signal that drives NK cell exhaustion [95,96]. Exhausted NKs show reduced degranulation (CD107a), granzyme B, and IFN-γ production, undermining their antiviral efficacy [96,97].

In the chronic inflamed microenvironment, dysfunctional NK cells contribute to a permissive niche for oncogenesis. Loss of NK-mediated elimination of damaged or transformed cells allows accumulation of genomic mutations and unchecked proliferation [98,99]. Moreover, exhausted NKs secrete lower levels of IFN-γ and CXCL10, diminishing recruitment and priming of CD8+ T lymphocytes. Persistent low-grade inflammation, driven by viral proteins and compromised NK function fosters the production of reactive oxygen species and pro-angiogenic factors, promoting DNA damage, extracellular matrix remodeling, and angiogenesis, which are hallmarks of early tumor initiation [95,99,100].

CD4+ helper T cells differentiate into specialized subsets (Th1, Th2, Th17, Tfh, Treg), each defined by distinct transcriptional programs and cytokine profiles that modulate the immune environment. Contrarily, Tregs secrete IL-10 and TGF-β, tempering excessive immune activation; however, in chronic infections such as HTLV-1 infection, this inadvertently fosters immunosuppressive niches that facilitate viral persistence and attenuate antitumor surveillance [101,102].

Sustained antigen exposure during chronic infection drives CD8+ cytotoxic T lymphocyte exhaustion, hallmarked by a progressive loss of effector functions, upregulation of inhibitory receptors (PD-1, CTLA-4, LAG-3, TIM-3), and altered metabolic programs favoring fatty acid oxidation over glycolysis [103]. Exhausted CTLs exhibit diminished proliferation and cytokine secretion, undermining their capacity to surveil and eradicate neoplastic clones. Viral oncoproteins further subvert CTL function by downregulating MHC class I on target cells or by inducing regulatory pathways that limit co-stimulation, thereby promoting immune escape and enabling the accumulation of DNA damage in infected tissues, which promotes virus-mediated carcinogenesis [103,104,105].

## 4. Epidemiology of Virus-Induced Human Cancers

Viral infections play a significant role in oncogenesis and are responsible for a considerable proportion of cancer cases worldwide. According to global estimates, approximately 15–20% of all human cancers are attributed to viral infections, representing a substantial impact on population morbidity and mortality [3,106]. The most studied oncogenic viruses include HPV, HBV and HCV, EBV, HTLV-1, and HHV-8. The prevalence and incidence of these infections vary globally, being influenced by geographic, socioeconomic, and behavioral factors and the availability of prevention programs, such as vaccines and population screening.

### 4.1. HPV and Its Relationship with Cancer

HPV is one of the most important oncogenic viruses, responsible for approximately 5% of all human cancers and about 99% of cervical cancer cases [6]. HPV infection is widespread, with more than 600 million individuals infected worldwide. HPV-16 and HPV-18 types are responsible for about 70% of cervical cancer cases, in addition to contributing to cancers of the anus, vulva, vagina, penis, and oropharynx [107].

HPV prevalence varies globally, reaching rates above 25% among sexually active young women in countries in Asia, Africa, and Latin America, where screening and vaccination programs do not yet cover the entire target population [107]. In developed countries, the incidence of cervical cancer has decreased significantly since the implementation of the HPV vaccine, with a low prevalence of infection in vaccinated populations. However, the incidence of HPV-associated oropharyngeal cancer has increased, especially in Western countries, due to changes in sexual habits and a greater number of lifetime sexual partners [108].

In terms of mortality, cervical cancer remains a major public health problem, causing around 342,000 deaths annually, with 85% of deaths occurring in low- and middle-income countries, where screening programs are scarce [109].

### 4.2. HBV, HCV, and Hepatocellular Carcinoma

HBV and HCV are directly implicated in hepatocellular carcinoma (HCC), one of the most lethal types of cancer. HBV infects more than 296 million people worldwide, while HCV infects approximately 58 million people [110]. The risk of progression to HCC in patients with a chronic HBV infection is 15–25 times higher than in uninfected individuals, and it is estimated that approximately 50% to 55% of global HCC cases are attributed to HBV, while 15–25% of cases are attributed to HCV [111].

The incidence of HBV- and HCV-associated HCC varies markedly geographically. The highest rates of HBV infection are observed in Asia and sub-Saharan Africa, regions where maternal-fetal transmission of the virus is common. China alone accounts for more than 50% of global cases of hepatocellular carcinoma [112]. HCV, on the other hand, is more common in Egypt, Eastern Europe, and the USA, and is transmitted primarily through injection drug use and inadequately sterilized medical procedures.

Since the advent of the hepatitis B vaccine, the global prevalence of chronic HBV infection has declined, especially among children vaccinated at birth. However, HCV still poses a significant challenge, as there is no vaccine available and many cases remain asymptomatic for decades, making early diagnosis and treatment difficult [113].

### 4.3. EBV and Lymphomas

EBV is associated with several types of cancer, including Burkitt’s lymphoma, Hodgkin’s lymphoma, and nasopharyngeal carcinoma. EBV infection is extremely common, affecting approximately 90–95% of the adult population worldwide [114].

The incidence of EBV-associated cancers varies across populations. Nasopharyngeal carcinoma is highly prevalent in Southeast Asia and southern China, with an incidence of 25–50 cases per 100,000 population in some regions, while in Western countries, it is a rare neoplasm [115]. Burkitt’s lymphoma, in turn, is more prevalent in sub-Saharan Africa, where co-infection with chronic malaria favors the proliferation of EBV and increases the risk of oncogenesis [116].

The role of EBV in oncogenesis has gained prominence due to its ability to persist in latent form in B lymphocytes, and can be reactivated under certain conditions, such as in immunocompromised patients, especially in transplant recipients or in carriers of human immunodeficiency virus (HIV)/acquired immunodeficiency syndrome (AIDS) [115].

### 4.4. HTLV-1 and Adult T-Cell Leukemia/Lymphoma

HTLV-1 is a retrovirus that infects approximately 10 million people worldwide, but its distribution is highly restricted to endemic regions such as southwestern Japan, Central Australia, South America, the Caribbean islands, and sub-Saharan Africa. Studies conducted in areas of high endemicity, such as sub-Saharan Africa, demonstrated a global prevalence of 1.67% (95% CI: 1.00–2.50%), while in regions of the Caribbean and South America, a seroprevalence of up to 3–5% was observed in local outbreaks, but the actual number is likely much higher due to incomplete data in other highly populated regions [117,118]. HTLV-1 infection can lead to the development of ATLL, an aggressive disease associated with short survival after diagnosis [119]. Japan, especially the Kyushu region, has the highest rates of HTLV-1 infection, with a prevalence of 5–10% of the adult population [120]. The main route of transmission of the virus is through breastfeeding, blood transfusions, and unprotected sexual contact.

The basic leucine zipper (HBZ) gene has been associated with the production of the inflammatory process in HTLV infection that culminates in the development of cancer. The induction of IFN-γ (interferon-gamma) production promotes chronic systemic inflammation, suggesting that IFN-γ and HBZ promote tumorigenesis in HTLV-1 infection [121].

### 4.5. HHV-8 and Kaposi’s Sarcoma

HHV-8 is the main cause of Kaposi’s sarcoma (KS), a vascular neoplasm that affects the skin and internal organs, especially in immunosuppressed individuals. The prevalence of HHV-8 is low in Western populations but reaches high rates in some countries in sub-Saharan Africa, where it can exceed 50% of the population, and is a major cause of HIV/AIDS-related cancer [122]. In recent years, the incidence of Kaposi’s sarcoma has declined in countries with widespread HIV antiretroviral therapy coverage but remains high in less developed regions where access to treatment is limited.

### 4.6. Merkel Cell Polyomavirus Associated with Merkel Cell Carcinoma

MCPyV is a widespread virus in the general population. Serological studies indicate that MCPyV infection is quite common, with a prevalence ranging from 60% to 80% in healthy adults, depending on the geographic region [123,124]. Primary infection usually occurs in childhood and is asymptomatic.

The virus can remain latent in the skin and other tissues throughout life. Although MCPyV infection is common, the development of MCC is rare. MCC is a highly aggressive neuroendocrine skin cancer with a mortality rate that can reach 33% within five years of diagnosis [124]. The incidence of MCC has increased in recent years, possibly due to an aging population and increased detection and diagnosis.

The global incidence of MCC is estimated to be about 0.7 cases per 100,000 people per year, but this rate varies significantly between different regions and populations. In the United States, the incidence of MCC is approximately 0.6 cases per 100,000 people per year, with about 2500 new cases diagnosed annually [125]. In Europe, the incidence ranges from 0.13 to 0.79 cases per 100,000 people per year, with the highest rates observed in northern countries such as Sweden and Norway [126].

## 5. Possible New Associations Between Viruses and Cancer

Research into the relationship between viral infections and cancer has revealed new associations and mechanisms by which viruses may contribute to oncogenesis. While some of these associations are well established, others are emerging with new scientific evidence.

### 5.1. Trichodysplasia Spinulosa Cell Polyomavirus (TSPyV) and Its Association with Skin Cancer

TSPyV was first identified in 2010 in immunocompromised patients with trichodysplasia spinulosa, a rare skin disease. Recent studies suggest a possible association between TSPyV and skin cancer. In a case report study, the presence of TSPyV DNA was detected in non-melanoma skin cancer samples, suggesting a potential role in cutaneous carcinogenesis [127,128]. However, as this is a case report, the study presents inherent limitations in its methodological robustness and does not provide conclusive evidence directly linking TSPyV to cancer development. Its primary focus lies in trichodysplasia spinulosa as a disease caused by TSPyV infection. Consequently, assertions regarding a potential association between TSPyV and oncogenesis remain limited. Further studies are warranted to elucidate the pathogenesis of trichodysplasia spinulosa, including comparative analyses between TSPyV infection and that of other classical human polyomaviruses (HPyVs) in order to investigate the oncogenic potential of TSPyV [128].

### 5.2. WU (WUPyV) and KI (KIPyV) Cell Polyomavirus and Their Association with Lung Cancer

WUPyV and KIPyV were discovered in 2007 and are associated with respiratory infections in immunocompromised children and adults. Recent studies have investigated the presence of these viruses in lung cancer samples. In a molecular analysis, WUPyV and KIPyV DNA were detected in a significant proportion of lung carcinoma samples, suggesting a possible association with pulmonary oncogenesis [129].

### 5.3. John Cunningham Cell Polyomavirus (JCPyV) and Its Association with Colorectal Cancer

JCPyV is known to cause progressive multifocal leukoencephalopathy (PML) in immunocompromised patients. Research has demonstrated a possible association between JCPyV and colorectal cancer. In molecular biology studies, JCPyV DNA has been detected in colorectal adenocarcinoma samples, and expression of the viral T-antigen protein has been observed in tumor cells, indicating a possible role in colorectal oncogenesis [130,131].

JCPyV genetic material and T antigen were also found in samples of adenoid cystic carcinomas of the trachea, paranasal sinuses, and oral cavity [132]. JCPyV DNA was detected more frequently in esophageal carcinomas than in normal, benign, or premalignant esophageal samples [133]. The JCPyV T antigen load is also higher in gastric cancer than that typically found in normal mucosa [134].

Although viral DNA and T-antigen protein have been reported in colorectal adenocarcinomas, the lack of matched normal samples and the absence of functional in vivo studies prevent us from distinguishing whether JCPyV drives neoplastic transformation or is only detected without causality in inflamed or preneoplastic tissues [132,133,134].

### 5.4. Hepatitis E Virus (HEV) and Its Association with Hepatocellular Carcinoma

Hepatitis E virus (HEV) is known to cause acute hepatitis, mainly in endemic regions. Recent studies have suggested a possible association between chronic HEV infection and hepatocellular carcinoma (HCC). In immunocompromised patients, such as those undergoing organ transplantation, chronic HEV infection has been associated with persistent hepatic inflammation and development of HCC [135]. Recently, a meta-analysis demonstrated the association of hepatitis E virus infection with an increased risk of hepatocellular carcinoma, however, well-designed clinical and experimental studies are needed to confirm these findings [136].

### 5.5. Bovine Papillomavirus (BPV) and Its Association with Bladder Cancer in Humans

BPV is known to cause papillomas and cancers in cattle, suggesting a possible association between BPV and bladder cancer in humans. In rural areas where contact with cattle is common, BPV DNA has been detected in human bladder cancer samples, suggesting possible zoonotic transmission and a role in oncogenesis [137].

### 5.6. Hepatitis G Virus (HGV/GBV-C) and Its Association with Non-Hodgkin’s Lymphoma

HGV/GBV-C is a flavivirus related to HCV. Recent studies have investigated the possible association between HGV/GBV-C and non-Hodgkin’s lymphoma. In a cohort of patients with lymphoma, the presence of HGV/GBV-C RNA was detected in a significant proportion of cases, suggesting a possible association with lymphoproliferative oncogenesis [138]. HCV is thought to increase the risk of NHL through chronic host infection and long-term immune stimulation [54,70]. Although direct effects on B cells are possible, GBV-C infection is thought to be a risk factor for non-Hodgkin’s lymphoma [139]. While studies contain evidence suggesting an association between an active GBV-C infection and increased risk of NHL, especially the diffuse large B-cell subtype, limitations related to the sample size of positive individuals, lack of standardized testing, and difficulty in assessing the persistence of infection and fully excluding other confounding factors indicate the need for further studies to confirm this association and fully understand its role in the etiology of NHL [138,139].

### 5.7. Torque Teno Virus (TTV) and Its Association with Hepatocellular Carcinoma

TTV is a small, circular DNA virus that infects humans and is widely disseminated in the population. Some studies have suggested a possible association between TTV and HCC. In patients with HCC, a high viral load of TTV has been observed, suggesting a possible contribution to hepatic oncogenesis. However, limitations due to this being a single case report prevent inferences about prevalence or causality in larger populations. The detection of TTV being restricted to serum with no evidence of viral DNA in tumor tissue or the adjacent liver tissue and in titers below the quantitative limit (<2 × 10^3^ copies/mL) raises doubts about its direct involvement in carcinoma formation [140,141].

Although several recent studies point to new correlations between viruses and cancer, the robustness of these findings varies widely and deserves critical analysis. In most investigations, the evidence is limited to the detection of viral nucleic acid in tumor samples that is almost always in small cohorts (*n* < 50); is without matched controls or adjustments for confounding factors such as immune status, concomitant infections, or chronic inflammatory processes (such as the associations with non-melanoma skin cancer and lung carcinoma with other polyomaviruses); and comes from too limited a case series, without validation of oncoprotein activity or animal models demonstrating transformation [127,128,129,130,131,132,133].

Thereby, reports linking these viruses to hepatocellular carcinoma, bladder cancer, and lymphomas are based almost exclusively on PCR of archived tissue, without viral load data, without stratification by chronic infection status, and without demonstration of molecular mechanisms (e.g., viral integration, interaction of viral proteins with p53/Rb). The need for prospective investigations is reiterated, with well-characterized positive and negative cohorts, and the analysis of specific genotypes to clarify whether it acts as a true tumor “driver” or only as a bystander in a predisposed hepatic environment [135,140].

## 6. Challenges

The association between oncoviruses and cancer development has revealed important insights into the mechanisms of oncogenesis. However, this area of study faces several challenges that directly impact the complete understanding of the role of oncogenic viruses in carcinogenesis. The discovery of new oncogenic viruses is a complex process that involves the identification of epidemiological associations, the detection of viral genetic material in tumor tissues, and the demonstration of causality. One of the main challenges is the confirmation that a newly identified virus is indeed oncogenic.

Studies of TSPyV and its possible association with skin cancer face the challenge of confirming causality. Although TSPyV DNA has been detected in non-melanoma skin cancer samples, evidence of causality is still limited [128]. Despite years of association between EBV and cancer, understanding the molecular mechanisms by which the virus promotes oncogenesis in different types of cancer, such as Burkitt’s lymphoma and nasopharyngeal carcinoma, is challenging due to the diversity of viral proteins involved and their multiple interactions with host cellular pathways [142].

The genetic variability of viruses and the genetic diversity of human populations pose significant challenges in research on oncogenic viruses. Different viral strains may have different oncogenic capabilities, and cancer susceptibility may vary between populations. The genetic diversity of HPV and varied prevalence in different regions make universal prevention strategies challenging. In addition, susceptibility to cervical cancer may vary between populations due to genetic differences and environmental factors [143].

The limitations of in vitro and in vivo models in reproducing complex biological environments and interactions between viruses and host cells have also emerged as a challenging area. For example, the development of animal models to study HBV-induced oncogenesis is complicated by the difference in the immune response between humans and animals. Transgenic mouse models have been used but do not fully replicate the pathogenesis of chronic HBV infection in humans [144].

## 7. Treatment

The high capacity of immune evasion and immunosuppression of oncoviruses is the subject of a challenging discussion for understanding the mechanisms involved in carcinogenesis, and it is crucial for developing effective therapies. Despite these viruses, some therapies have been established to prevent the development of cancer resulting from the infectious process (Table 3).

Vaccination against HPV and HBV represents one of the most effective preventive strategies against virus-induced cancers, as these are the only oncoviruses for which licensed prophylactic vaccines exist. HPV can be prevented by vaccines that elicit durable immunity against types 16 and 18, its primary oncogenic strains. HBV, the etiological agent of hepatocellular carcinoma, can likewise be prevented by administering a single birth-dose vaccine followed by two or three additional doses, ensuring long-term protection and markedly reducing both the incidence of chronic infection and the risk of hepatocellular carcinoma development [145,154,155].

However, vaccination efforts face critical barriers in low- and middle-income countries, including budgetary constraints, gaps in cold-chain infrastructure, and limited community acceptance—exacerbated by poor awareness of the virus–cancer link and vaccine safety myths—as well as logistical challenges posed by multi-dose schedules and shortcomings in political commitment and service integration [156,157].

## 8. Perspective

The relentless pursuit of advances in biotechnology, immunotherapy, and genomics are opening new perspectives for preventing, diagnosing, and treating cancers associated with oncogenic viruses.

Vaccination has been one of the most effective strategies in preventing oncogenic viral infections. Scientific studies have shown promising results, with experimental vaccines inducing robust immune responses that may be promising for some viruses such as EBV and HTLV-1 [158,159]. In addition to prophylactic vaccines, there is growing interest in the development of therapeutic vaccines that can treat chronic viral infections and associated cancers. Therapeutic HPV vaccines are being developed to treat precancerous lesions and established cancers. Early clinical studies show that these vaccines can induce specific immune responses against HPV-infected cells, leading to the regression of lesions [160].

Although HTLV-1 was identified nearly four decades ago, there are still no vaccines approved for human use. Although research efforts have intensified in recent years, no vaccine candidate is currently in clinical trials. Several approaches have been investigated in preclinical models, including viral vector-based vaccines (such as vaccinia and adenovirus), DNA vaccines, immunogenic synthetic protein or peptide vaccines, and, more recently, mRNA platforms encapsulated in lipid nanoparticles. These strategies have shown the ability to induce neutralizing antibodies, activation of cytotoxic CD8+ T cells, and, in some cases, significant reduction in proviral load or protection against infection in animal models [161].

However, development faces substantial challenges, such as the complexity of the viral life cycle, which includes persistent infection of CD4+ T lymphocytes and cell-to-cell transmission, making it difficult to induce effective immunity. Furthermore, there is a need for combined neutralizing antibody and T-cell responses, which requires sophisticated immunization protocols. Additional barriers include the limited structural understanding of the viral envelope glycoprotein gp46, continuing suboptimal immune responses to some formulations, and the scarcity of clinical trials validating safety and efficacy in humans [161].

The development of new antivirals is crucial for the treatment of chronic viral infections and the prevention of progression to cancer, as are other therapeutic alternatives with immunotherapy, including immune checkpoint inhibitors and chimeric antigen receptor T-cell (CAR-T) therapies that are revolutionizing the treatment of cancers associated with oncogenic viruses [162,163].

New technologies, such as CRISPR-Cas9 gene editing, offer new possibilities for treating chronic viral infections and preventing viral oncogenesis. Gene editing can be used to interrupt viral replication or correct genetic mutations associated with cancer [164]. CRISPR-Cas9 has been used to edit HBV and HCV genes in liver cells, interrupting viral replication and preventing progression to HCC [165,166].

Advanced approaches using CRISPR-Cas9-based editing variants enable the introduction of precise point mutations such as premature stop codons into viral genes, inhibiting their expression without inducing double-strand breaks in the host DNA, which significantly reduces the risk of genomic instability. Several delivery platforms have been developed to maximize the efficiency and specificity of this approach, including adeno-associated virus (AAV) vectors, magnetic nanoparticles, and hepatocyte-targeted delivery systems, with the aim of minimizing off-target effects [167,168,169,170].

Experimental results demonstrate that CRISPR-Cas9-mediated editing promotes substantial reductions in viral gene expression, HBV DNA levels, and surface antigens, both in cell cultures and in animal models. Complete excision of integrated viral DNA and destabilization of cccDNA has led to long-lasting suppression, or even eradication, of viral markers in cell lines. However, important challenges remain. Efficient delivery of the CRISPR system to infected cells, ensuring absolute specificity for viral targets, and minimizing unwanted genomic alterations remain technical hurdles to overcome [169,170].

In the context of infectious processes, the global implementation of vaccination programs is one of the key points of prevention and requires the strengthening of the health system. Notably, the implementation of vaccination is a key point for reducing the viral load of infections and the transmissibility of viruses. We observe this scenario in the reduction of the incidence of HPV infections due to the availability of vaccines for the population, which directly impacts the reduction of cervical cancer and other neoplasms associated with the virus [145]. The WHO emphasizes the need to implement HPV vaccination programs in low- and middle-income countries where the burden of cervical cancer is highest. Therefore, strengthening health systems to improve early diagnosis, population-based screening, prophylaxis, and treatment of oncogenic viral infections is a global priority. Screening programs for cervical cancer and viral hepatitis are being expanded in several regions of the world with the support of international organizations and local governments. These programs aim to increase screening coverage and improve access to treatment for vulnerable populations [110].

Although significant advances have been made in understanding viral oncogenesis, several questions remain unresolved and warrant future investigation. One promising area concerns the role of co-infections, such as the association between HIV-1 and HHV-8 in the development of Kaposi’s sarcoma. Co-infection with HIV-1 and HHV-8 generates a sustained systemic immune activation, which is crucial to the pathogenesis of Kaposi’s sarcoma (KS) [171]. Co-infected individuals exhibit elevated levels of soluble immune-activation markers—including sCD163, sCD25/IL-2Rα, and sCD40/TNFRSF5—that correlate positively with HIV viral load and negatively with CD4+ T-cell counts. Even under effective antiretroviral therapy, systemic inflammation remains higher than in HIV-negative patients [171].

Virus-specific immune dysfunction exacerbates this scenario, as CD8+ and CD4+ T-cell responses against HHV-8 antigens are frequently deficient in both AIDS-associated and classic KS patients, permitting HHV-8 reactivation from latency [172]. Inflammatory cytokines—particularly IFN-γ—can trigger viral reactivation in infected cells, leading to viremia and viral dissemination. Viral evasion strategies, such as the K3 and K5 proteins that downregulate antigen-presentation molecules, and vFLIP, which inhibits FAS-mediated apoptosis, impair recognition and clearance by T and NK cells [173].

Furthermore, the HIV-1 Tat protein acts as a tumor-promoting cofactor: secreted within KS lesions, Tat stimulates proliferation, migration, and invasion of tumor-derived endothelial cells, synergizing with HHV-8 vGPCR to activate the NF-AT and NF-κB pathways. Local production of angiogenic factors—such as VEGF and acidic FGF—is elevated in co-infected individuals, correlating with immune-activation markers and contributing to the angioproliferative phenotype of KS [173,174].

These multifaceted interactions between HIV-1 and HHV-8 exemplify the viral synergies that drive oncogenesis, creating an environment conducive to the reactivation and proliferation of latent oncoviruses and amplifying their transforming potential. However, a detailed understanding of the specific molecular mechanisms underlying these interactions remains incomplete and is essential for developing targeted interventions that interrupt the cycle of immune activation and viral reactivation in co-infected patients [174].

Another emerging field is the impact of the human microbiome on viral carcinogenesis. Evidence shows that the host’s microbial community significantly impacts viral oncogenesis through interactions among bacteria, viruses, and host cells [175]. Recent studies suggest that microbial composition and diversity can modulate immune responses, affect viral persistence, and even interact directly with viral oncogenes, thereby altering the risk of cellular transformation [175].

Directly, bacterial components or their metabolites, such as lipopolysaccharides, short-chain fatty acids, and other fermentation products, can influence virion infectivity and stability [176]. For example, bacterial LPS enhances the structural resilience of certain enteroviruses, while fatty acids produced by gut commensals have been implicated in reactivating latent oncoviruses like EBV and KSHV, thereby restarting the lytic cycle in previously quiescent cells [177,178,179,180].

Concurrently, the microbiota shapes the host’s immunological milieu, indirectly influencing susceptibility to virus-driven cancers. Bacteria such as *Helicobacter hepaticus* drive chronic hepatic inflammation by persistently activating the NF-κB pathway, which synergizes with HCV infection to accelerate progression to hepatocellular carcinoma [181]. Likewise, *Chlamydia trachomatis* in the cervical epithelium amplifies pro-tumorigenic NF-κB signaling and elevates survival and angiogenic factors (VEGF, survivin) in HPV-infected cells [182].

Site-specific studies further underscore this complexity. In the oral microbiome of Kaposi’s sarcoma patients, reduced diversity and an overrepresentation of *Firmicutes* correlate with higher HHV-8 loads and immune-activation markers, suggesting that dysbiosis promotes both viral reactivation and the angioproliferative phenotype [183]. Conversely, beneficial genera such as *Bifidobacterium* can enhance antiviral and antitumor immunity by stimulating NK and CD8+ T-cell responses [184].

Both direct interactions, such as virion stabilization and reactivation, and indirect effects, including inflammation and immune modulation, highlight virus–bacteria–oncogenesis interactions as a promising area for identifying potential therapeutic targets [184]. Modulating the microbiota—particularly within the gut and tumor microenvironment—may also influence responses to cancer therapies, including chemotherapy and immune checkpoint blockade (PD-1, CTLA-4) [185,186]. Despite the progress made, significant challenges persist. There is still no universal consensus on whether any specific cancer requires a bacterium–virus axis for carcinogenesis. Furthermore, the complex interactions between host, virus, and bacteria necessitate additional mechanistic studies to elucidate the precise molecular pathways involved [184,187].

## 9. Conclusions

A detailed understanding of the molecular mechanisms of viral oncogenesis is critical to develop effective preventive strategies (vaccination), advanced antiviral treatments, innovative immunotherapies, and microbiome-modulation-based interventions to reduce the global burden of virus-related cancers.

The principal mechanisms of oncogenesis include integration of viral genomes into host DNA, expression of oncoproteins that inactivate p53 and RB, disruption of cellular signaling pathways, induction of oxidative stress, and epigenetic modulation. It illustrates how these viral strategies drive uncontrolled proliferation, immune evasion, and establishment of a permissive tumor microenvironment, underscoring the need to dissect each molecular event for the development of targeted interventions.

This review summarizes key strategies for cancer prevention and treatment, including prophylactic vaccines, antiviral therapies, immunotherapies, and CRISPR/Cas9 methods, underscoring the importance of conducting studies that investigate the dynamics of co-infection and the role of the microbiota in viral persistence and its ability to cause cancer, which will certainly open new possibilities for combined therapeutics and microbiome-modulating interventions to prevent the progression of virus-associated tumors.

## Figures and Tables

**Table 1:** Viruses with oncogenic potential for cancer development.

Virus Name	Genome	Viral Family	Cancer	Transmission	Reference
*Papillomavirus Humano* (HPV)	dsDNA	*Papillomaviridae*	Penile, throat, cervical, vaginal, and vulvar cancer	Sexual contact, skin-to-skin contact, skin-to-mucosa contact, mother-to-baby contact, and contaminated fomites	[7]
Epstein–Barr virus (EBV)	dsDNA	*Herpesviridae*	Burkitt’s lymphoma, B-cell lymphoma, nasopharyngeal carcinoma, and gastric cancer	Body fluids (genital, blood)	[8]
Kaposi’s sarcoma herpesvirus (KSHV)	dsDNA	*Herpesviridae*	Kaposi’s sarcoma	Saliva and genital secretions	[9]
Hepatitis C virus (HCV)	sRNA	*Flaviviridae*	Hepatocellular carcinoma and non-Hodgkin’s lymphoma	Blood and body fluids	[10]
Hepatitis B virus (HBV)	dsDNA	*Hepadnaviridae*	Hepatocellular carcinoma	Blood and body fluids	[10]
Human T-lymphotropic virus 1 (HTLV-1)	sRNA	*Retroviridae*	Adult T-cell leukemia	Body fluids (semen, breast milk)	[11]
Merkel cell polyomavirus (MCV)	dsDNA	*Polyomaviridae*	Merkel cell carcinoma	No validated route	[12]

Abbreviations: ds, double-stranded; ss, single-stranded.

**Table 2:** The main oncoviruses and their mechanisms of oncogenesis.

Virus	Oncoprotein	Site of Action	Mechanism of Oncogenesis
HPV	E6, E7	p53, pRb	Degradation of tumor suppressors Integration into the genome
EBV (HHV-4)	LMP1, EBNA	Cell signaling receptors	Immortalization of B lymphocytes Integration into the genome
HBV	HBx	Signal transduction pathways	Stimulates cell proliferation Integration into the genome
HCV	Core, NS5A	Mitochondria, signaling pathways	Induces chronic inflammation Angiogenesis Inhibition of apoptosis
KSHV (HHV-8)	LANA, vFLIP	Tumor suppressors	Inhibition of apoptosis
HTLV-1	Tax, HBZ	JAK/STAT, NF-κB	Activates lymphocyte proliferation Integration into the genome Chronic inflammation
MCV	LT, sT	Cell cycle	Induces uncontrolled proliferation

**Table 3:** Therapeutic evidence implemented for the treatment of oncogenic viruses.

Oncogenic Virus	Associated Cancer	Treatments	Evidence	Reference
HPV (human papillomavirus)	Cervical, oropharyngeal, anal, vulvar, and penile cancer	Prophylactic vaccination: Gardasil Cervical treatment of precancerous lesions: LEEP, cryotherapy, laser treatment	Vaccination: 83% reduction in the prevalence of HPV-16 and HPV-18 infections in vaccinated girls	[145]
HBV (hepatitis B virus)	Hepatocellular carcinoma (HCC)	Antiviral therapies: Tenofovir, Entecavir, Pegylated Interferon (Peg-IFN)	Antiviral: significant reduction in HBV viral load and incidence of HCC	[146]
HCV (hepatitis C virus)	Hepatocellular carcinoma (HCC)	Antiviral therapies: DAAs (Sofosbuvir, Ledipasvir, Daclatasvir)	DAAs: cure rates greater than 95%, reduced risk of cirrhosis and HCC	[147]
EBV (Epstein–Barr virus)	Burkitt’s lymphoma, Hodgkin’s lymphoma, nasopharyngeal carcinoma	Antiviral Therapies: Acyclovir Valganciclovir immunotherapy: Pembrolizumab, Nivolumab	Antivirals: limited reduction in viral load, limited impact on cancer prevention Immunotherapy: durable responses in EBV-associated lymphomas	[148,149]
HTLV-1 (human T-lymphotropic virus 1)	Adult T-Cell leukemia/lymphoma (ATLL)	Antiviral Therapies: Zidovudine Interferon-alpha targeted therapies: Bortezomib, Vorinostat	Antivirals: improved survival in patients with ATLL Targeted therapies: induction of apoptosis in HTLV-1-infected cells	[150,151]
HHV-8 (human herpesvirus type 8)	Kaposi’s sarcoma	Antiviral therapies: combined ART	ART: reduction in the incidence and progression of Kaposi’s sarcoma in patients with HIV/AIDS	[152]
MCPyV (Merkel cell polyomavirus)	Merkel cell carcinoma (MCC)	Immunotherapy: Avelumab, Pembrolizumab	Immunotherapy: long-lasting responses in patients with MCC, improved survival	[153]

## Data Availability

No new data were created or analyzed in this study. Data sharing is not applicable to this article.

## Supplementary Materials

The following supporting information can be downloaded at: https://www.mdpi.com/article/10.3390/biology14070797/s1, Figure S1: PRISMA flow diagram. Reference [188] are cited in the supplementary materials.

## Abbreviations

The following abbreviations are used in this manuscript:

EBV	Epstein-Barr virus (EBV)
HPV	human papillomavirus (HPV)
HBV	hepatitis B virus
HCV	hepatitis C virus
KSHV	Kaposi’s sarcoma-associated herpesvirus
HHV-8	human herpesvirus 8
HTLV-1	human T-cell lymphotropic virus
MCPyV	Merkel cell polyomavirus
IARC	International Agency for Research on Cancer
OV	oncoviruses
ATLL	adult T-cell leukemia/lymphoma
CYPs	cytochrome P450 enzymes
MPC	mitochondrial pyruvate transporter
TME	tumor microenvironment
CAFs	cancer-associated fibroblasts
CDKs	cyclins and cyclin-dependent kinases
DNMTs	E-cadherin through overexpression of DNA methyltransferases
ROS	reactive oxygen species
NF-κB	nuclear factor kappa B
HIV/AIDS	human immunodeficiency virus (HIV)/acquired immunodeficiency syndrome (AIDS)
HBZ	leucine zipper
KS	Kaposi’s sarcoma
HEV	hepatitis E Virus
BPV	bovine papillomavirus
TTV	torque teno virus
HGV/GBV-C	hepatitis G virus
CAR-T	chimeric antigen receptor T-cell
HCC	hepatocellular carcinoma
