# Supplementary material for: The Impact of Oncogenic Viruses on Cancer Development: A Narrative Review
Source: Biology (Basel). 2025 Jul 1;14(7):797. doi: 10.3390/biology14070797 (PMC12292244; doi:10.3390/biology14070797)
Supplement: Supplementary file 1 [file biology-14-00797-s001.zip › biology-3689522-supplementary.pdf]

Flow Diagrame PRISMA

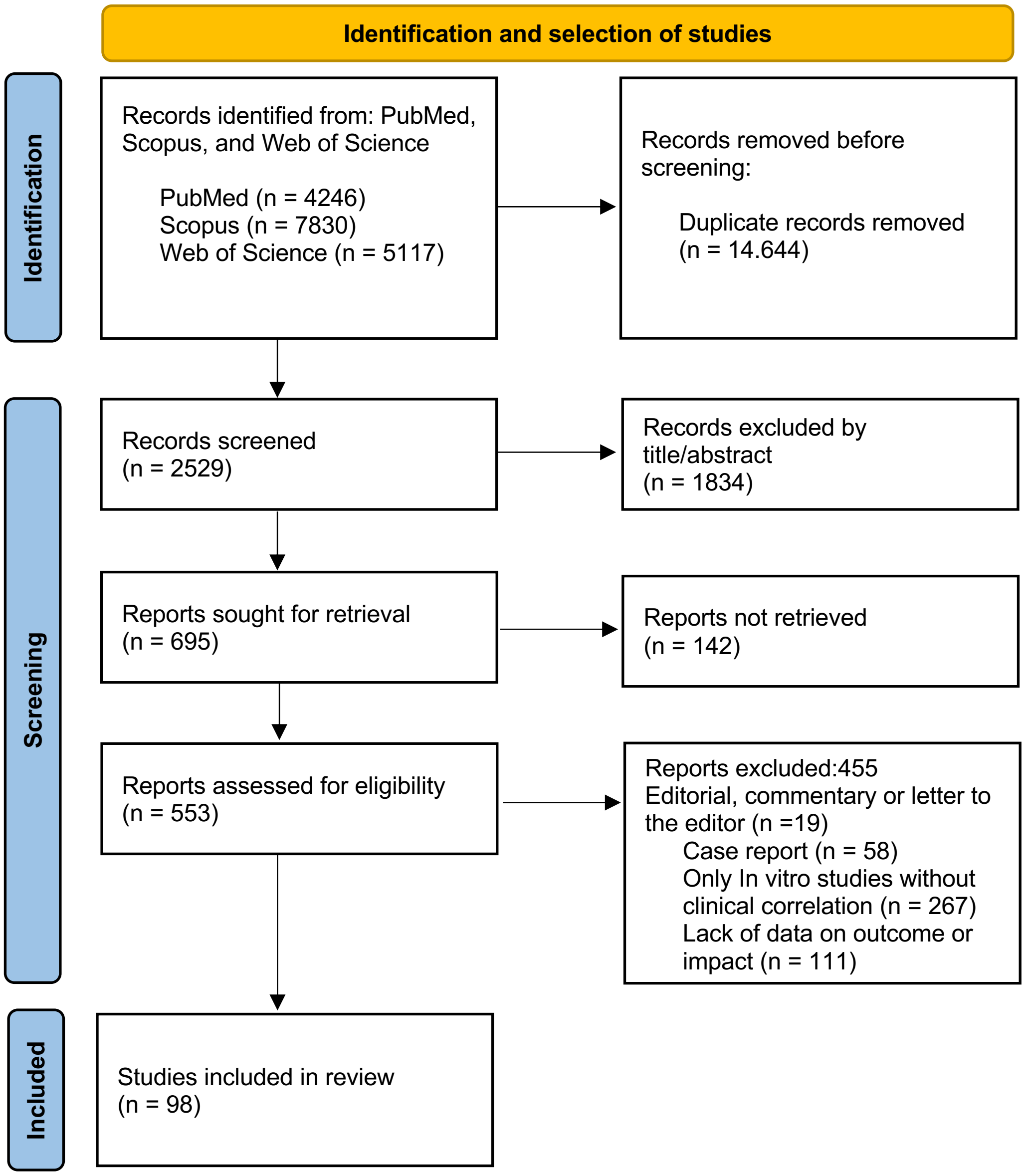

Source: Page MJ, et al. BMJ 2021;372:n71. doi: 10.1136/bmj.n71.

This work is licensed under CC BY 4.0. To view a copy of this license, visit https://creativecommons.org/licenses/by/4.0/
